# Abnormal microglial polarization induced by *Arid1a* deletion leads to neuronal differentiation deficits

**DOI:** 10.1111/cpr.13314

**Published:** 2022-07-19

**Authors:** Maolei Gong, Ruoxi Shi, Yijun Liu, Jinpeng Ke, Xiao Liu, Hong‐Zhen Du, Chang‐Mei Liu

**Affiliations:** ^1^ State Key Laboratory of Stem Cell and Reproductive Biology, Institute of Zoology Chinese Academy of Sciences Beijing China; ^2^ Institute for Stem Cell and Regeneration Chinese Academy of Sciences Beijing China; ^3^ Beijing Institute for Stem Cell and Regenerative Medicine Beijing China; ^4^ Savaid Medical School University of Chinese Academy of Sciences Beijing China

## Abstract

**Objective:**

Microglia, the prototypical innate immune cells of the central nervous system (CNS), are highly plastic and assume their phenotypes dependent on intrinsically genetic, epigenetic regulation or extrinsically microenvironmental cues. Microglia has been recognized as key regulators of neural stem/progenitor cells (NSPCs) and brain functions. Chromatin accessibility is implicated in immune cell development and functional regulation. However, it is still unknown whether and how chromatin remodelling regulates the phenotypic plasticity of microglia and exerts what kind of effects on NSPCs.

**Methods:**

We investigated the role of chromatin accessibility in microglia by deleting chromatin remodelling gene *Arid1a* using microglia‐specific Cx3cr1‐cre and Cx3cr1‐CreERT2 mice. RNA‐seq and ATAC‐seq were performed to dissect the molecular mechanisms. In addition, we examined postnatal M1/M2 microglia polarization and analysed neuronal differentiation of NSPCs. Finally, we tested the effects of microglial *Arid1a* deletion on mouse behaviours.

**Results:**

Increased chromatin accessibility upon *Arid1a* ablation resulted in enhanced M1 microglial polarization and weakened M2 polarization, which led to abnormal neurogenesis and anxiety‐like behaviours. Switching the polarization state under IL4 stimulation could rescue abnormal neurogenesis, supporting an essential role for chromatin remodeler ARID1A in balancing microglial polarization and brain functions.

**Conclusions:**

Our study identifies ARID1A as a central regulator of microglia polarization, establishing a mechanistic link between chromatin remodelling, neurogenesis and mouse behaviours, and highlights the potential development of innovative therapeutics exploiting the innate regenerative capacity of the nervous system.

## Graphical abstract

Microglia are thought to play an essential role in differentiation and maturation of neural precursor cells into neurons. As the prototypical innate immune cells of the central nervous system (CNS), the balance of microglial polarization is regulated by genetic or epigenetic modulators. Our study showed enhancement of chromatin accessibility by the chromatin remodelling gene *Arid1a* deletion led to abnormal development of microglia, which shows heightened M1 microglial polarization and weakened M2 polarization. In addition, irregular polarization led to abnormal neurogenesis with a significant decrease of neural stem/progenitor cell differentiation into neurons.

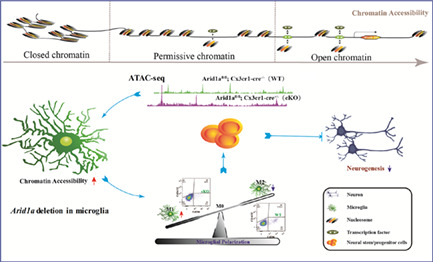

## 1 INTRODUCTION

Microglia, the only resident immune cells that enduringly reside in the central nervous system (CNS),^1^ play vital roles in regulating the homeostasis of CNS.^2^ In the past years, studies on microglia have expanded from investigating their immunological functions as resident macrophages of brain and mediators of neuroinflammation, injury, neurodegeneration to understanding their origins and functions involved in complex neurodevelopmental programs in the CNS.^3^, ^4^, ^5^, ^6^ Evidence has shown that microglia modulate neurogenesis and synaptic pruning, during which they interact with neural stem/progenitor cells (NSPCs) or neurons to provide trophic support, respond to cytokines and drive the refinement of differentiation or maturation of precursors into neurons.^7^ The role of microglia appears to be a double‐edged sword in the battle for CNS damage and neurological recovery. Microglia activation acts as defender of the brain by resolving local inflammation, clearing cell debris, and releasing trophic factors^8^, ^9^ whereas microglia activation can also incumber neurogenesis and exacerbate tissue damage.^10^, ^11^, ^12^ However, how to control or balance microglia activation and its subsequent effects on other cells in CNS are largely unknown.

Recent research has shown that microglia can harbour different phenotypes depending on intrinsic and extrinsic signals. Like microphages, microglia have three typical phenotypes, including two polarization types (M1 and M2) and a resting type (M0).^13^ Under different stimulus, microglia could be activated into M1 and M2 polarized states. However, the mechanisms leading to their activation toward a specific phenotype and the reversibility of microglia polarization are not yet fully established. Microglia are involved in many brain disorders; however, little is known about how intrinsic or extrinsic signals impact microglial phenotype switch and function.

Chromatin remodelling, modulated by chromatin remodelers, is the rearrangement of chromatin from a condensed state to a transcriptionally accessible state, allowing transcription factors or other DNA‐binding proteins to access DNA and control gene expression.^14^ As a kind of M1 microglia inducer, LPS can switch microphage/microglia polarization. Gene loci analysis of LPS‐inducible genes reveals particular requirements for SWI/SNF‐dependent nucleosome remodelling in the transformation of microglia polarization.^15^, ^16^ The evolutionarily conserved multi‐protein SWI/SNF complex, one of the largest chromatin‐remodelling complexes, plays a critical role in coordinating chromatin architecture and gene expression.^17^ Importantly, emerging remodelling in chromatin landscape, particularly enhancer landscape, plays an important role in macrophage polarization.^18^, ^19^, ^20^ ARID1A, the largest subunit of the multi‐protein SWI/SNF chromatin remodelling complex,^21^ assembles with either BRM or BRG1 to form a functional chromatin‐remodelling complex.^22^ *Arid1a* has a dominant role in maintaining chromatin accessibility at enhancers,^23^ acting as an important regulator of most essential neurogenic genes during early neural development.^24^ Notably, *Arid1a* mutation is correlated with altered expression of various genes and gene sets involved in the immune response,^25^ which indicates that *Arid1a* may have function in immune cells. Human genetic studies have identified dysregulation of ARID1A in Coffin‐Siris syndrome (CSS), a congenital disorder characterized by intellectual disability and growth deficiency.^26^ Furthermore, early‐life inflammation and the potential roles for microglia have been documented in immune‐driven animal models of autism and schizophrenia.^27^ These studies, together with our previous data showing that *Arid1a* has a high expression in microglia cells in CNS,^28^ suggesting a potential role for *Arid1a* in microglia and inflammation phenotypes observed in neurodevelopmental disorders. However, there are no animal models available to study the direct role for Arid1a in the modulation of microglia phenotypes.

Here, we established a conditional allele to manipulate cell‐type‐specific *Arid1a* function in CNS microglia. We found that *Arid1a* loss in microglia prior to birth led to abnormal polarization, which then resulted in enhanced M1 microglial polarization and weakened M2 polarization. Importantly, microglial polarization anomaly induced by absence of *Arid1a* led to abnormal neurogenesis and anxiety‐like behaviour. We further identified microglia M2 phenotype inducer interleukin‐4 (IL‐4) could stimulate the switch of microglia polarization and restored abnormal neurogenesis. Collectively, our results demonstrate a critical role for ARID1A in the development of microglia and neuronal differentiation, which provides insights into the genetic basis and potential therapeutic interventions of ARID1A‐related intellectual disabilities such as Coffin–Siris syndrome.

## 2 MATERIALS AND METHODS

### 2.1 Mice

All animal experiments were approved by Laboratory Animal Center, Institute of Zoology, Chinese Academy of Sciences, and were performed in accordance with the respective national regulations of China. The *Arid1a* ^fl/fl^ mouse^29^ was a kind gift from Dr Zhong Wang (Harvard Medical School). To generate microglia‐specific Arid1a deleted animals, we crossed *Arid1a* ^fl/fl^ mouse with either Cx3cr1‐Cre (JAX Stock No. 005628) or Cx3cr1‐CreERT2 (JAX Stock No. 020940) line. To get the iKO mice line, 8 mg of Tamoxifen (TAM, Sigma‐Aldrich) dissolved in corn oil was applied by intraperitoneal injections to mice from P0 to P5. All mice were bred in specific‐pathogen‐free facility with a 12 h light/dark cycle. Mice genotypes were performed by PCR assay using tail genomic DNA. Genotyping primers are listed in Table S2 (Supplementary Information). All procedures were approved by Animal Committee of Institute of Zoology, Chinese Academy of Sciences.

### 2.2 Primary cell culture

For NSPCs, forebrain from P7 pups was freed from meninges and washed in phosphate buffer saline (PBS) with gentle trituration followed by centrifugation at 400*g* for 10 min. Next, brain tissues were digested with TrypLEME Express (Gibco, 12,604,013) at 37°C for 10 min. Neurobasal™ Medium containing 10% FBS (Gibco, 10,099,141), 1% GlutaMAX (Gibco, 35,050,061) and 1% Penicillin–Streptomycin (Hyclone, SV30010) was added into each digestion product to stop digestion. After repetitive pipetting, cell suspension was passed through 70‐μm cell strainer, and then was cultured with Neurobasal™ Medium containing N2 supplement (0.5%, GIBCO) and B27 (0.5%, GIBCO), Penicillin–Streptomycin (1%, Hyclone), fibroblast growth factor‐2 (20 ng/ml, PeproTech), and epidermal growth factor (20 ng/ml, PeproTech) in a 5% CO_2_ incubator at 37°C. For differentiation of NSCs, NSCs were first seeded on poly‐l‐ornithine/laminin‐coated coverslips at a density of 1 × 105 cells/well. NSCs were incubated with DMEM/F‐12 medium containing N2 supplement and B27, Penicillin–Streptomycin, forskolin (5 μM, Sigma‐Aldrich), and retinoic acid (5 μM, Sigma‐Aldrich) for 3 days.

For microglia, cortical hemispheres from newborn mice (P0‐3) were freed from meninges, and digested with TrypLEME Express at 37°C for 10 min. And then, cell suspension was plated in 25 cm^2^ tissue culture flasks with DMEM/F12 supplemented with 10% FBS. After a 2 weeks culture, the flasks were shaken for 2 h at 130 rpm to harvest microglia. For microglia and NSPCs co‐culture, 2000 microglia were seeded on a single well of 24‐well plate for 24 h, 50 ng/mL Il4 was added if needed, following with 10^5^ NSPCs planting. When the secretion function was done, 2000 microglia was seeded over 0.4 μm trans well, and 10^5^ NSCs blow.

### 2.3 BrdU incorporation analysis

Based on the mouse weight, intraperitoneal injection was used to inject pregnant mice at day 18.5 with 100 mg/kg BrdU (Sigma, B5002‐5G). The brains were harvested at P7 and P14 for the following analysis.

### 2.4 Flow cytometry

Mice were anaesthetized with avertin (sigma) and transcardially perfused with ice‐cold PBS. The tissue was digested using a Papain Dissociation System kit (Worthington, LK003150) following the manufacturer's recommendations. Cell suspension was collected and washed extensively with fluorescence activated cell sorter (FACS) buffer (2% FBS in PBS) followed by staining with anti‐CD11b APC (clone M1/70, Biolegend) and anti‐CD45 PE (clone 30‐F11, Biolegend) for microglia isolation, and anti‐mouse CD16/32 APC/cy7 (clone 93, Biolegend) and anti‐mouse CD206 FITC (clone c068c2, Biolegend) for polarization analysis.

### 2.5 RNA‐seq, ATAC‐seq and ChIP‐seq analysis

The total RNA was extracted from FACS sorted WT or cKO microglia according to procedures with TRIzol reagent (Invitrogen, 15596018). A 2 μg RNA was used for sequencing libraries generation using NEBNext® Ultra™ RNA Library Prep Kit for Illumina® (#E7530L, NEB, USA) following the manufacturer's recommendations. The cDNA library was analysed by Illumina HiSeq 2500 platform. After that, the library was measured using Qubit® RNA Assay Kit in Qubit® 3.0 to preliminary quantify and then dilute to 1 ng/μl. Insert size was assessed using the Agilent Bioanalyzer 2100 system (Agilent Technologies, CA, USA), and qualified insert size was accurate quantification using StepOnePlus™ RT PCR. The clustering of the index‐coded samples was performed on HiSeq PE Cluster Kit v4‐cBot‐HS (Illumina), and the libraries were sequenced on an Illumina platform. Finally, 150 bp paired‐end reads were generated with help of Annoroad Gene Tech. (Beijing) Co., Ltd. The RNA‐seq data are available in Sequence Read Archive (SRA) under BioProject PRJNA812340.

For ATAC‐seq, 50,000 primary microglia were taken for library preparation. The cells were lysed in Lysis Buffer (10 mM NaCl, 3 mM MgCl2, 0.15% NP‐40, in pH 7.410 mM Tris–HCl) to get the nuclei, and TruePrep™ DNA Library Prep Kit V2 for Illumina (Vazyme Biotech, TD501) was used to construct the transposase‐treated libraries. Qubit 3.0 Fluorometer and StepOnePlus™ real‐time PCR system used for the mass concentration and molar concentration of libraries analysis, respectively. Agilent HS 2100 Bioanalyzer was used to detect the lengths of inserted fragments. Qualified libraries were sequenced by Illumina HiSeq X 10 platform in pair‐end 150 bp style with the help of Annoroad Gene Tech. (Beijing) Co., Ltd. The ATAC‐seq data are available in SRA under BioProject PRJNA812630.

For ChIP‐seq, the Chip‐seq data for *Arid1a* was downloaded from SRA under BioProject PRJNA638150.^30^

### 2.6 Western blot analysis and RT‐PCR

Total RNA from FACS sorted or primary microglia was reverse transcribed into cDNA using Transcript One‐Step gDNA Removal and cDNA synthesis Kit (TransGen Biotech, Beijing, China). Then, cDNA was quantified by using Hieff® qPCR SYBR® Green Master Mix (Yeasen Biotech, 11201ES08). The analysis of relative gene expression was executed by the 2^−ΔΔCT^ method, and actin was used as endogenous control. RT‐PCR primers are listed in Table S1 (Supplementary Information).

The total protein of primary microglia was extracted using RIPA buffer containing 10 mM PMSF (Beyotime Biotechnology, ST505), and the concentration of it was defined using BCA protein assay kit (Beyotime Biotechnology, P0012). The membranes were blocked in 5% milk in TBS‐T (0.1% Tween 20) and incubated with the primary antibodies, including Anti‐beta Tubulin (1:5000, Abcam) and anti‐Arid1a (1:2000, Sigma) at 4°C overnight. The membranes were then washed in TBS‐T for 10 min × 3 times, and then incubated with the secondary antibodies at room temperature for 2 h. ECL system (Pierce) and Tanon‐5200 Chemiluminescent Imaging System (Tanon, China, Shanghai) was used for signal detection conducting.

### 2.7 Immunofluorescence staining

The brain isolated from perfused mice was post‐fixed with 4% paraformaldehyde (PFA) overnight, and dehydrated with 30% sucrose later. The tissue was used for cryo embedding, and cut into 35‐μm‐thick cryosections. For primary cells, cultured cells were seeded on coverslips and fixed with PFA. To get immunofluorescence analysis results, sagittal brain slices or coverslips were permeabilized (0.5% Triton X‐100, 3% BSA in PBS) for 15 min and blocked (0.3% Triton X‐100, 3% BSA in PBS) for 1 h at room temperature, first. Next, slices were incubated with the relevant primary antibodies (0.3% Triton X‐100, 3% BSA in PBS) overnight at 4°C, including anti‐Arid1a (1:500, Sigma), anti‐Iba1 (1:500, Wako), anti‐Tuj1 (1:1000, Biolegend), anti‐GFAP (1:500, Abcam), anti‐BrdU (1:500, Abcam), anti‐NeuN (1: 1000, Millipore) and Anti‐Doublecortin (1:500, Abcam). Alexa Fluor 488, 568, 594 or 647 secondary antibodies (1:500, Life Technologies) were used for corresponding staining, and Nuclei were counterstained with DAPI (1:1000, Sigma).

### 2.8 Microscope imaging and three‐dimensional reconstruction of microglia

Confocal images were acquired using Zeiss LSM880 confocal microscopes and analysed by ZEN software. Three‐dimensional (3D) surfaces were reconstructed using the IMARIS surface reconstruction tool depending on Iba1 immunofluorescence staining.

### 2.9 Behaviour tests

#### 2.9.1 Open field

Open‐field testing was conducted using 6–8 –week‐old male mice in a 33 cm × 33 cm × 40 cm box for 15 min, and total distance, average speed, time spend in the centre, and entrance times of the centre were quantified by Smart 3.0 during the first 10 min. The centre zone was marked as a 14 cm square.

#### 2.9.2 Light–dark box testing

The light–dark box (LDB) was made of white and black opaque Plexiglas (20 cm × 30 cm × 30 cm light chamber, 30 cm × 30 cm × 30 cm dark chamber). The chambers were connected by a 10 × 10 cm door in the middle of the wall separating the two chambers. Animals were placed in the middle of the dark box for 3 min facing a side away from the door and then released. LDB was conducted for 8 min with time in the light box and light box entries calculated with Smart 3.0 during the first 5 min. Mice were used as in Open field.

#### 2.9.3 Elevated plus maze

Elevated plus‐maze testing was conducted for 8 min with time in the open arm and open arm entries calculated with Smart 3.0 during the first 5 min. Mice were used as in Open field.

#### 2.9.4 Morris water maze test

A circular water tank (diameter = 120 cm) was filled with water, and the water was made opaque with non‐toxic white paint. A round platform (diameter = 13 cm) was hidden 1 cm beneath the surface of the water at the centre of a given quadrant of the water tank. Mice received training in the Morris water maze for 6 successive days; each session consisted of 4 trials. A probe test was conducted 24 h after completion of the training. During the probe test, the platform was removed from the pool, and task performance was recorded for 60 s.

#### 2.9.5 Fear conditioning test

Prior to the experiment, mice were handled for 5 min per day for 3 consecutive days. During training, mice explored context A (grid floor with transparent cage walls) for 2 min, then received one pairing of a tone (amplitude 85 dB for 30 s) co‐terminated with a foot shock (0.6 mA, 1 s). Contextual fear was tested 24 h after conditioning by placing the animals in the conditioning context (context A) for a 5‐min period. For auditory fear conditioning, the test consisted of a 2‐min acclimatizing period to the context B (grid floor and cage walls were covered with yellow plastic to create another context), followed by a 3‐min period during which the tone was delivered (CS).

#### 2.9.6 Marble burying test

A large cage (45 cm × 22 cm) with transparent filter tops filled with 5 cm of fresh and compact corncob bedding and 4 × 6 marbles (diameter = 2.3 cm) that were equally distributed along the walls. Mice were placed in the middle of the cage and left undisturbed for 30 min. Finally, the number of marbles, which more than 2/3 of their volume was buried, was counted. Mice were used as in Open field.

#### 2.9.7 Self‐grooming test

The self‐grooming task consisted of an initial 10 min of habituation, followed immediately by a 10 min measure of self‐grooming. Mice were singly placed in plastic containers (45 cm × 22 cm). Grooming time was recorded in seconds, and self‐grooming behaviour included obvious self‐grooming on genital, tail, paw, leg, body and head.

### 2.10 Statistical analysis

Data are represented as mean ± SEM, unless otherwise indicated. Experiments were conducted in at least 3 biological replicates (*n* ≥ 3) for each group. For statistical analyses, unpaired Student's *t*‐tests were executed using GraphPad Prism software. Statistical significance was defined as *p < 0.05, **p < 0.01, ***p < 0.001, ****p < 0.0001.

## 3 RESULTS

### 3.1 *Arid1a* ablation enhances microglial chromatin accessibility and alters transcriptomic profile

**FIGURE 1:**
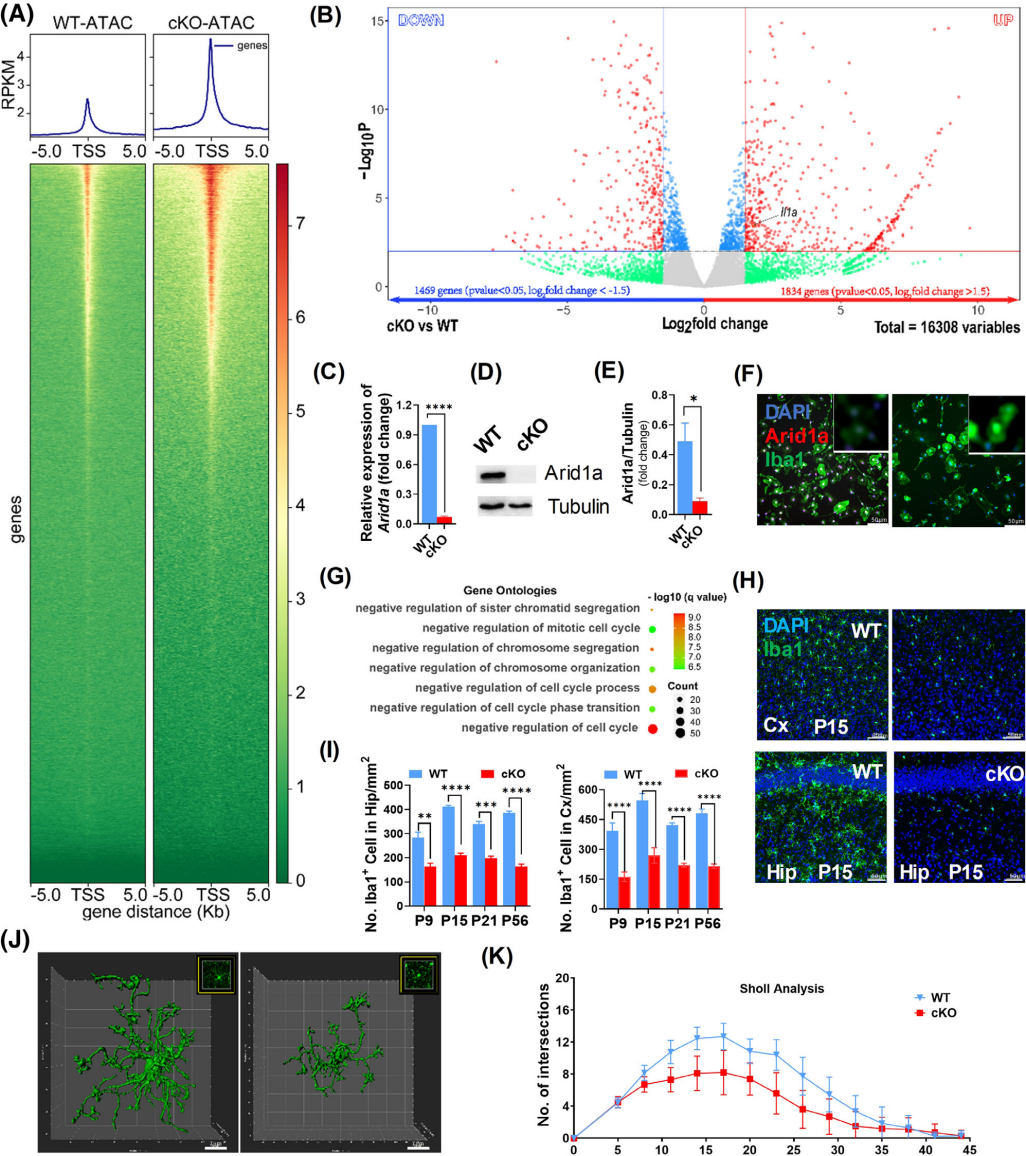
Absence of *Arid1a* Alters Microglial Chromatin Accessibility, Transcriptomic Profile and Development. (A) Average profile of ATAC‐seq signal in *Arid1a* cKO microglia compared to WT, plotted within +/− 5 kb of transcription start site (TSS, down) and fragments per kilo base of transcript per million mapped fragments (PRKM, up) of NCBI‐annotated genes. (B) Example volcano plot. Points on top‐right and top‐left corners are considered the most promising genes (*p* < 0.05, log2foldchange >1.5) in 16,308 significantly changed genes. (C–E) Expression of *Arid1a* in primary microglia cells. Total RNAs (C) and protein (D) were extracted from WT and cKO primary microglia and tested with RT‐PCR and western blotting and the expression of Arid1a protein level was quantified in (E). (F) Arid1a staining in cultured primary microglia isolated from WT and cKO. (G) Pathway analysis of cell cycle‐related affection based on gene ontology (GO) database. Count means number of genes involved in the specific pathway. (H–I). Representative image (H) and Quantification (I) of microglia in cortex and hippocampus. Iba1 was used for frozen section staining collected at day 9, 15, 21, 56 after birth. (J, K) Morphology of microglia in WT and cKO. Three‐dimensional (3D) surfaces of microglia were reconstructed using the IMARIS surface reconstruction tool depending on Iba1 immunofluorescence staining (J), and the sholl analysis was analysed using ImageJ and the results were shown in (K). Data are expressed as mean ± SEM and analysed by Student's *t*‐test from at least three independent experiments. *p < 0.05, **p < 0.01, ***p < 0.01 and ****p < 0.01

The *Arid1a* gene, which encodes a subunit of the SWI/SNF chromatin remodelling complex, has an ARID domain considered to facilitate non‐specific DNA binding.^31^, ^32^ To study the function of *Arid1a* and assess the effects of *Arid1a* ablation on chromatin accessibility in microglia, we first generated *Arid1a* ^fl/fl^;Cx3cr1‐cre mice line by crossing *Arid1a* ^fl/fl^ with Cx3cr‐cre transgenic mice to delete *Arid1a* specifically within microglia in brain (Figure S1A). We isolated primary microglia from *Arid1a* ^*fl/fl*^;Cx3cr1‐cre^+^ (cKO) and the corresponding control *Arid1a* ^fl/fl^;Cx3cr1‐cre^−^ (WT) mice. Compared to WT, the absence of *Arid1a* in microglia was first validated by quantitative real‐time RT‐PCR (Figure 1C) and western blotting (Figure 1D, E). And similarly, cKO microglia has none localized ARID1A in nucleus as shown in immunostaining (Figure 1F), indicating that *Arid1a* was deleted.

We then analysed chromatin accessibility with ATAC‐seq in primary *Arid1a* cKO and WT microglia. Our results showed that cKO microglia dramatically altered overall chromatin accessibility compared to WT (Figure 1A and Figure S1G). The alterration of chromatin accessibility in microglia impaired transcriptomic profile of microglia isolated from cKO as compared with WT based on microglial RNA‐seq analysis (Figure 1B).

### 3.2 Enhanced chromatin accessibility in microglia lacking *Arid1a* results in microglial malformation

Enrichment analysis supported strong up‐regulation of gene ontologies (GO) such as negative regulation of cell cycle (GO:0045786), negative regulation of cell cycle process (GO:0010948), and negative regulation of chromosome segregation (GO:0051985, Figure 1G), which confirmed the results that *Arid1a* absence led to decreased proliferation of microglia. We then analysed the impact on microglia by using IBA1 as a microglia marker in the mouse hippocampus and cortex (Figure 1H). The quantification result showed that there was an increased number of cells expressing Iba1 in cKO mice compared to WT at postnatal day (P) 9, 14, 21 and 56 (Figure 1I). To confirm that, we then generated *Arid1a* ^fl/fl^;Cx3cr1‐creERT2 mice and induced *Arid1a* deletion with tamoxifen (TAM) injection starting from P0 (Figure S1B). The cortical and hippocampus microglia also demonstrated a significant decrease of cell number after TAM injection at P14 (Figure S1C–E). IMARIS‐based 3D morphometric measurements of microglia further revealed that microglia exhibited shorter processes and reduced numbers of terminal points at P14 (Figure 1J‐K). These data suggested that *Arid1a* is indispensable for proliferation and structural construction of microglia.

### 3.3 *Arid1a* absence results in enhanced M1 microglial polarization and decreased M2 polarization

**FIGURE 2:**
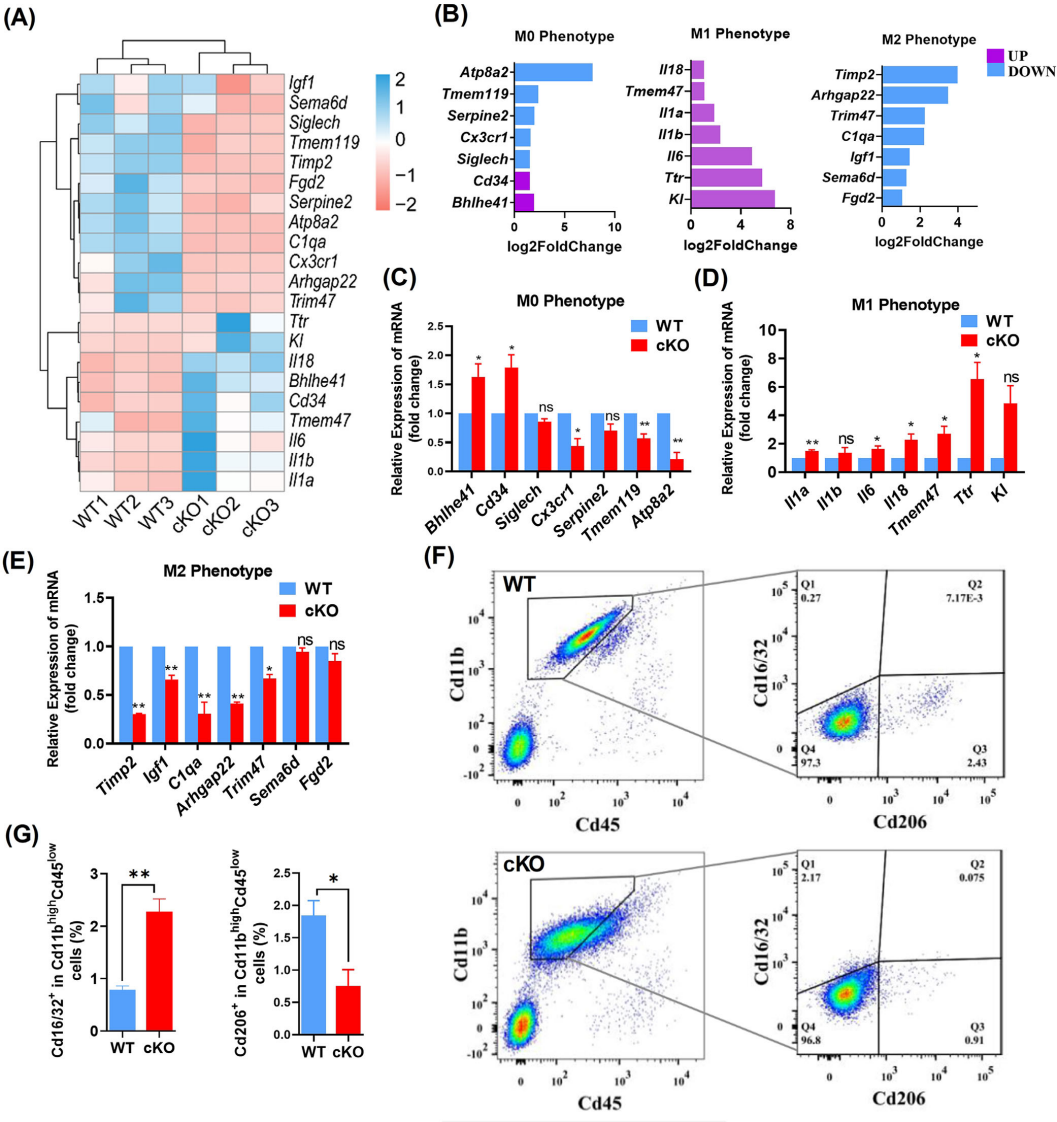
*Arid1a* absence results in enhanced M1 type polarization. **(**A) Hierarchical clustering of microglial polarization‐related genes which are significantly altered (*p* < 0.05, log2foldchange >1.5) in cKO microglia of P14 mice in RNA‐seq analysis (*N* = 3), compared to WT. The heatmap displays z‐transformed expression values. (B) Log2foldchange of microglial polarization‐related genes (*p* < 0.05), which are significantly changed between microglia from cKO and WT mouse at P14 obtained from RNA‐seq analysis. (C) Expression of M0 microglia marker genes. The expression of these genes was examined at mRNA levels using RT‐PCR, β‐actin was used as a standard control. Total RNA was extracted from cKO and WT microglia isolated through FACS. (D). Expression of M1 microglia marker genes. (E) Expression of M2 microglia marker genes. (F, G) Flow cytometric characterization of microglia in P14 mice. Cd45 PE and Cd11b APC antibodies were used for microglia identification, Cd16/32 APC/Cy7 and Cd206 FITC anti‐mouse specific antibodies were used for M1 or M2 microglial identification, respectively (F), the ratio of M1 or M2 in total microglia was analysed by the number of Cd16/32^+^ or Cd206^+^ cells per the number of Cd45^low^Cd11b^high^ cells (G). Data are expressed as mean ± SEM and analysed by Student's *t*‐test from at least three independent experiments, *p < 0.05 and **p < 0.01

Based on our RNA‐seq analysis, we found that *Il1a* expression increased significantly upon *Arid1a* deletion (Figure 1B, black arrow). The enhancement of *Il1a* expression is considered as a marker for microglia M1 polarization,^33^ suggesting that *Arid1a* ablation may result in abnormal microglia polarization. To directly assess the function of *Arid1a* in microglial polarization, we analysed the expression of microglial genes involved in polarization (Figure 2A, *padjust <* 0.05, log2foldchange >1.5). The RNA‐seq results showed that M1 microglial genes, including *Ki*, *Tlr*, *Il1a*, *Il1b*, *Il6*, *Il18* and *Tmem47* significantly increased (Figure 2B, middle). In contrast, M2 microglial genes, including *Igf1*, *C1qa*, *Arhgap22*, *Tim47*, *Timp2*, *sema6d* and *fgd2*, significantly decreased (Figure 2B, right). However, M0 microglial genes remained unchanged (Figure 2B, left). This gene expression pattern was confirmed further by qRT‐PCR (Figure 2C–E). Moreover, we used FACS analysis and identified the polarization states of microglia isolated from cKO and WT mice at P14 by M1/M2 labelled antibodies (Figure 2F). The results demonstrated that the ratio of Cd16/32 positive cells (M1) in Cd45^low^&Cd11b^high^ cells markedly increased (Figure 2G, left), but the ratio of Cd206 positive cells (M2) decreased (Figure 2G, right). Collectively, these results indicated that loss of *Arid1a* altered the balance of microglial M1/M2 polarization, suggesting an essential role for ARID1A in the phenotypic plasticity of microglia.

### 3.4 Enhanced chromatin accessibility in microglia lacking *Arid1a* dysregulates neurogenesis

**FIGURE 3:**
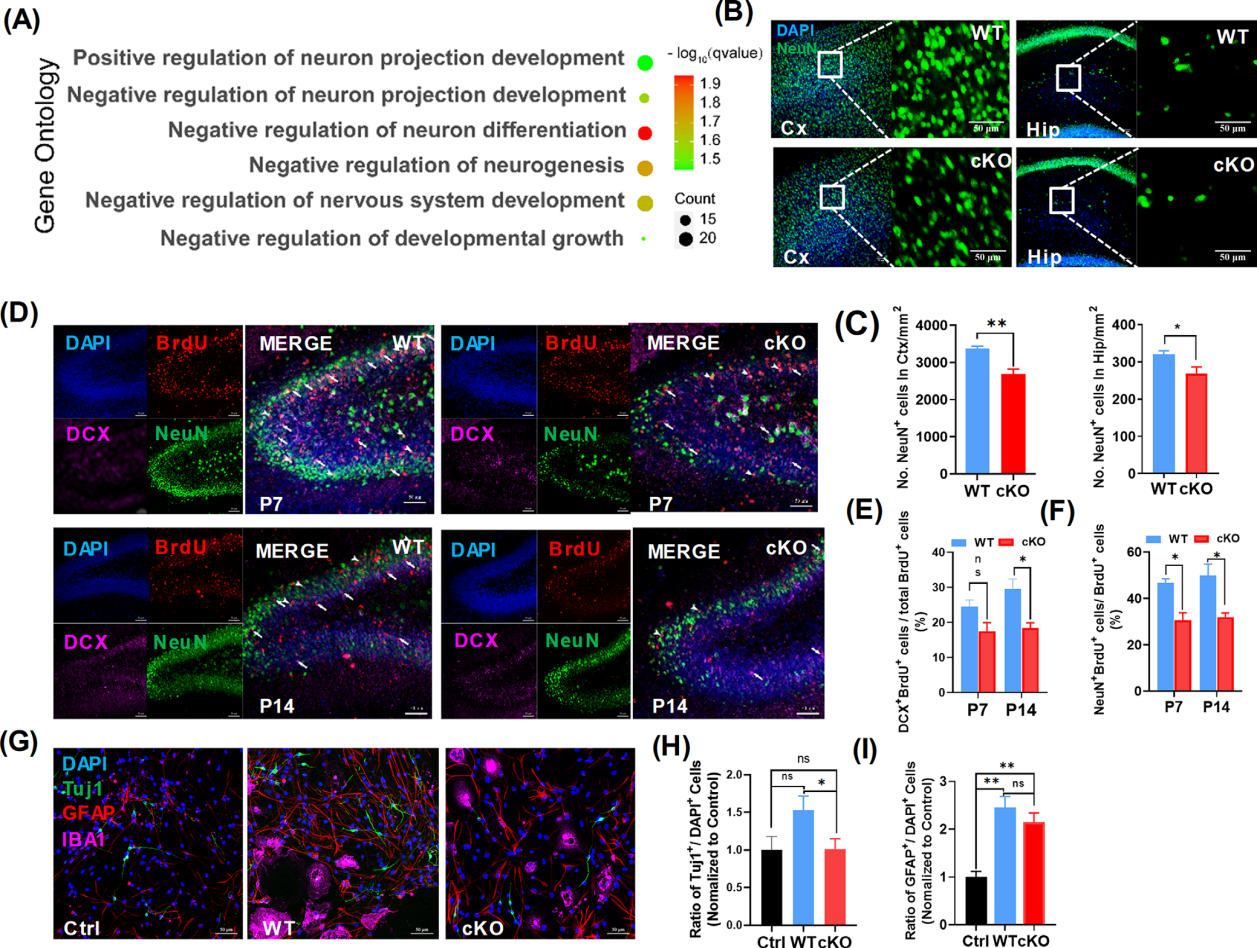
Absence of microglia *Arid1a* during development inhibits neuronal differentiation. (A) Pathway analysis of neuro‐related affection based on gene ontology (GO) database. Count means number of genes involved in the specific pathway. (B, C) Cortex and hippocampus NeuN staining in *Arid1a* cKO and WT mice. Immunofluorescence staining was performed in frozen section of 14‐day‐old mice using NeuN antibody (B), and the number of neurons in cortex and hippocampus (C) were quantified by NeuN^+^ cells per square millimetre, respectively. (D–F) Hippocampal DG area staining of *Arid1a* cKO and WT mouse. Immunofluorescence staining was performed in frozen section of 7‐ or 14‐day‐old mice using specific antibodies BrdU, DCX and NeuN (D), BrdU was injected at E18.5 and the number of neurons differentiated from neural stem/progenitor cells (NSPCs) were quantified by DCX^+^Brdu^+^ (E) or NeuN^+^Brdu^+^ (F) cells in BrdU^+^ cells. (G–I) Differentiation test of NSPCs in vitro. Total of 100,000 NSPCs from WT mice were co‐cultured with 20,000 cKO or WT microglia for NSPCs differentiation, specific antibodies for Iba1, Tuj1 and GFAP were used for analysis of NSPCs differentiation (G), and the percentage of differentiated neurons (H, Tuj1^+^ cells) and astrocyte (I, GFAP ^+^ cells) were showed. Scale bar = 50 μm. Data are expressed as mean ± SEM and analysed by Student's *t*‐test from at least three independent experiments, *p < 0.05 and **p < 0.01

Recent studies indicate that microglial polarization has important effects on neurogenesis,^10^, ^34^ during which M1 microglia inhibits and M2 microglia promotes neurogenesis.^35^ In our microglia‐specific *Arid1a* ablation mouse model, we found abnormal M1/M2 polarization, as well as alterations of gene expression were involved in neurogenesis. Based on GO enrichment, we found that *Arid1a* loss in microglia can affect neurons supported by significant up‐regulation of negative regulation of neuronal differentiation term (GO:0045665), and negative regulation of nervous system development (GO:0051961), indicating that microglial *Arid1a* ablation may cripple neurogenesis (Figure 3A).

To test the hypothesis that microglia polarization anomaly induced by *Arid1a* deletion may cripple neurogenesis, we first quantified differentiated neurons by NeuN immunofluorescence (Figure 3B). The results showed that the number of neurons markedly decreased in the cortex (Figure 3C, left) and hippocampus of cKO mice (Figure 3C, right). To investigate whether the decrease of neurons was caused by deficient neurogenesis upon *Arid1a* loss in microglia, we injected mice with BrdU at E18.5 to label proliferative NSPCs and then analysed the newly generated neurons after birth. BrdU, Doublecortin (DCX) and NeuN immunofluorescence staining was used to label the cell identity (Figure 3D). The quantification results showed that, compared to WT, both the immature neurons (BrdU^+^ DCX^+^) (Figure 3D, white arrows with tails) and mature neurons (BrdU^+^NeuN^+^) (Figure 3D, white arrows without tails) that differentiated from NSPCs dramatically decreased at P7 and P14 (Figure 3E, F). To further verify the defects of neurogenesis observed in cKO mice, we co‐cultured primary NSPCs with primary microglia isolated from P7 mice and analysed the differentiation abilities of NSPCs (Figure 3G). Compared to WT, NSPCs co‐cultured with cKO microglia differentiated into fewer neurons (Figure 3H), but astrocytes differentiation was not affected (Figure 3I). Moreover, compared to WT, NSPCs co‐cultured with cKO microglia did not alter proliferation efficiency of NSPCs (Figure S1I‐J), but enhanced cell apoptosis (Figure S1K‐L). Together, these data provide strong support of an essential role for ARID1A in maintaining the balance of microglia polarization and promoting neurogenesis.

### 3.5 *Arid1a* modulates Il‐1a expression and affects neuronal differentiation of NSPCs

**FIGURE 4:**
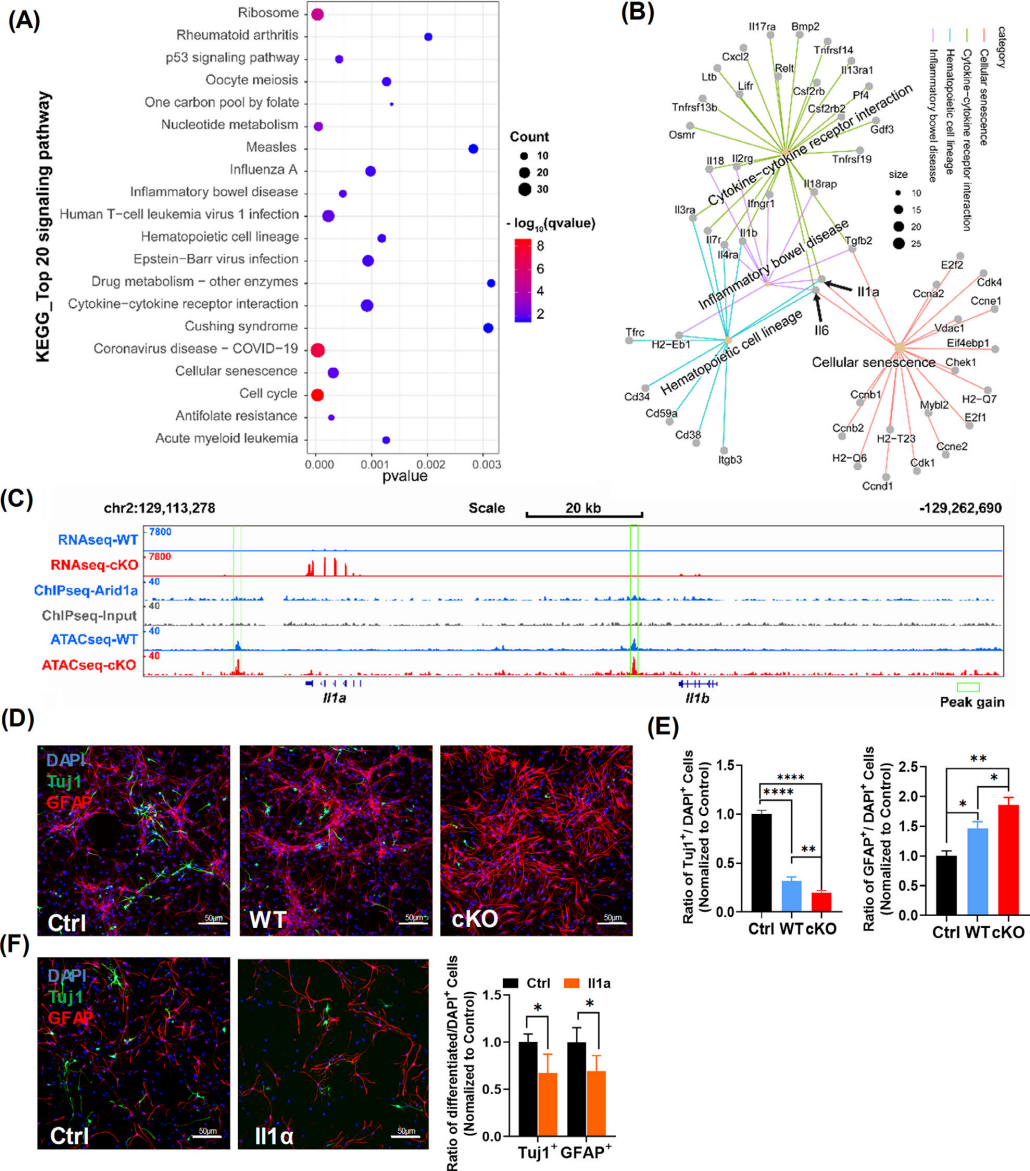
*Arid1a* Modulates Il1a Expression and Affects Neuronal Differentiation of neural stem/progenitor cells (NSPCs). **(**A) Top 20 pathway analysis based on KEGG database revealing the differentially expressed genes (*p* < 0.05, log2foldchange >1.5) in cKO microglia were enriched for multiple cellular biological processes, count means number of genes involved in the specific pathway. (B) Cellular secretion‐related KEGG pathway gene network analysis, including cytokine–cytokine receptor interaction, inflammatory bowel disease, cellular senescence and haematopoietic cell lineage. Size means number of genes involved in the specific pathway, the black arrow points to the genes involved in all these pathways above, including *Il6* and *Il1a*. (C). Genome‐browser view of RNA‐seq (*n* = 3), ChIP‐seq (*n* = 1), and ATAC‐seq (*n* = 2) at *Il1a* and *Il1b*, the green rectangle was used for pointing out the peak gain. (D) NSPCs differentiation using microglia and NSPCs contactless culture system and 0.4 μm trans well were used. For NSPCs differentiation, 20,000 cKO or WT microglia were seeded on the upper side of the trans well membrane and 100,000 NSCs from WT mice were seeded on the lower side, and specific antibodies for Tuj1 and GFAP were used for analysis of NSPCs differentiation. (E) Quantification of Tuj1and GFAP positive cells (*n* = 3). (F) Il1a‐stimulated NSPCs differentiation. Left, representative images of Tuj1 and GFAP staining. Right, quantification of Tuj1and GFAP positive cells (*n* = 3). Data are expressed as mean ± SEM and analysed by Student's *t*‐test from at least three independent experiments. *p < 0.05, **p < 0.01, and ****p < 0.0001

Phagocytosis and secretion are the most basic functions of microglia.^36^ To determine whether *Arid1a* ablation affects gene expression associated with the fundamental functions of microglia, such as phagocytosis and secretion, we performed top 20 kyoto encyclopaedia of genes and genomes (KEGG) analysis. The results revealed that the differentially up‐regulated genes (*padjust* <0.05, log2foldchange >1.5) in cKO microglia were enriched in 4 pathways related to cell secretion, including cytokine‐cytokine receptor interaction (mmu04060), inflammatory bowel disease (mmu05321), haematopoietic cell lineage (mmu04640) and cellular senescence (mmu04218, Figure 4A). Combined gene network analysis further demonstrated that *Il1a* (interleukin 1 alpha) and *Il6* (interleukin 6) were involved in all these 4 pathways (Figure 4B, black arrows). Genome‐browser view of ATAC‐seq showed a significant peak gain of *Il1a* (Figure 4C), but not *Il6* (Figure S1 H). To further analyse whether *Arid1a* could directly regulate *Il1a* expression，we downloaded the deposited *Arid1a* ChIP‐seq data.^30^ The analysed data demonstrated that ARID1A has enrichment on the site where *Il1a* showing a significant peak gain after *Arid1a* deletion in our ATAC‐seq, suggesting that ARID1A could directly regulate *Il1a* expression (Figure 4C). Taken together, these data indicated that microglial abnormality in the absence of *Arid1a* during development affected neuronal differentiation and cytokines secretion.

As we showed above, cytokines secretion may play an important role in ARID1A‐mediated functions in microglia (Figure 4A). Unlike cell‐to‐cell connections, paracrine does not need direct contact between cells. We then asked whether paracrine of microglia mediates the deficiency of *Arid1a* cKO NSPCs in neuronal differentiation. We established microglia and NSPCs contactless culture system with 0.4 μm trans well. The differentiation of NSPCs seeded on the lower side was analysed using immunofluorescent staining (Figure 4D). Compared to WT, cKO NSPCs significantly reduced neuronal differentiation (Figure 4E, left) and enhanced astrocyte differentiation (Figure 4E, right). Given that we observed a significant up‐regulation of gene expression and chromatin accessibility of *Il1a* in *Arid1a* cKO microglia, we then investigated whether ARID1A promotes neuronal differentiation through Il1a secretion. By directly adding recombinant mouse Il1a to the cultured primary NSPCs with neuronal differentiation medium (Figure 4F, left), we found that Il1a treatment decreased neuronal differentiation in cultured NSPCs (Figure 4F, right). These results indicated that *Arid1a* ablation in microglia causes neuronal differentiation defects likely through Il1a overexpression.

### 3.6 Il4‐stimulated switch of microglia polarization corrects neuronal differentiation defects

**FIGURE 5:**
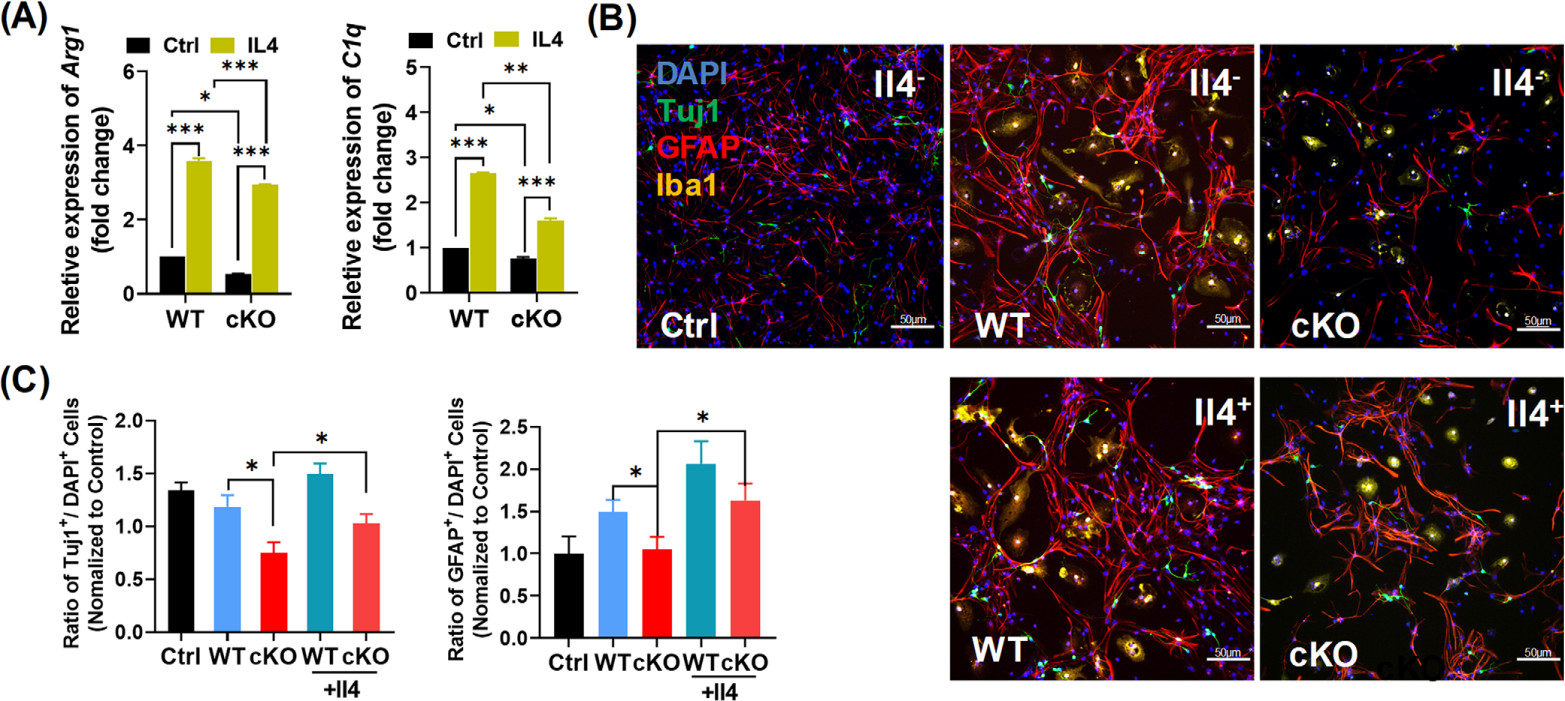
Switching microglia polarization corrects deficient neuronal differentiation. **(**A) The expression of Arg1 and C1q in WT and cKO microglia. Total RNA was extracted from Il4‐stimulated Microglia, actin was used as an internal reference. (B) Differentiation of neural stem/progenitor cells (NSPCs) co‐cultured with Il4‐stimulated microglia in vitro. Iba1, Tuj1 and GFAP were used for analysis of NSPCs differentiation. (C) The ratio of differentiated neurons (left) and astrocytes (right) were shown. Scale bar = 50 μm. Data are expressed as mean ± SEM and analysed by Student's t‐test from at least three independent experiments. *p < 0.05, **p < 0.01, ***p < 0.001

Last, we assessed whether switch of irregular microglia polarization from M1 to M2 could correct neuronal differentiation deficiency caused by *Arid1a* deletion in microglia. We used Il4, which has been demonstrated to stimulate M2 polarization,^35^ to culture microglia in vitro. RT‐PCR results showed significant enhancement of M2 microglial polarization with increased expression level of *Arg1* and *C1q*, markers of M2 microglia, under Il4 stimulation (Figure 5A). Moreover, compared to Il4 free group, Il4‐treated *Arid1a* cKO group demonstrated the recovery of neuronal differentiation (Figure 5B, C), which indicates that switching the abnormal microglial polarization states induced by *Arid1a* ablation in microglia is sufficient to reverse the deficiency of neuronal differentiation. Together, these findings demonstrate that the abnormal dynamic equilibrium of microglial polarization following *Arid1a* deletion is a key mechanism which mediates the defective neurogenesis and brain development in *Arid1a* cKO mice.

### 3.7 Absence of microglial *Arid1a* prior to birth leads to anxiety‐like behaviour in mice

**FIGURE 6:**
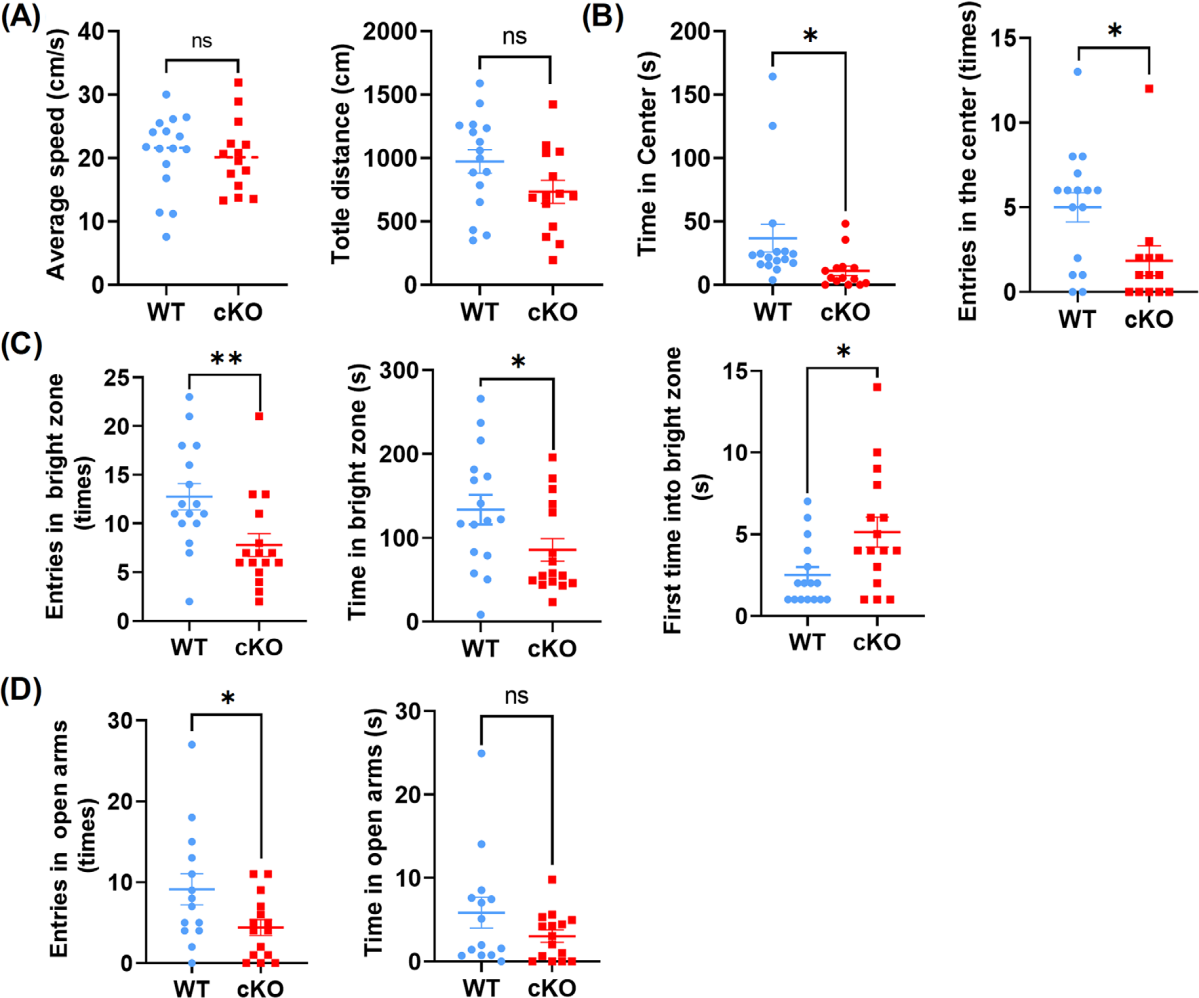
Microglial *Arid1a* Absence prior to birth leads to anxiety‐like behaviour in mice. (A, B) Open field test of *Arid1a* cKO mice and 6–8‐week‐old male mice (*n* > 14) were used for open field test, and the average speed (A, left), total distance (A, right), time in centre (B, right), and number of entries to the central area (B, left) were determined. (C) The light–dark transition test. Mice were used as in (A), the numbers of entries to the bright zone (left), the first time into the bright zone (middle), and time in the bright zone were shown (right). (D) Elevated plus maze. Mice were used as in (A), and the number of entries to the open arms (left) and the time spent in the open arms (right) were analysed. Data are expressed as mean ± SEM and analysed by Student's *t*‐test, *p < 0.05 and **p < 0.01

Previous studies indicate that neurogenesis has significant correlation with emotion, especially depression and anxiety.^37^, ^38^ Abnormal neurogenesis causes behavioural impairments, including learning and memory.^39^, ^40^ What's more, evidence shows that defects of immune cells, including CD4^+^ T‐cells, Th17/Treg, mast cell and microglia,^41^, ^42^, ^43^, ^44^ lead to mouse anxiety or anxiety‐like behaviours. We then assessed whether the deficient neurogenesis caused by microglial *Arid1a* deletion could affect mouse behaviours. As revealed in open‐field tests, although *Arid1a* cKO mice showed significant loss in body weight (Figure S2J), they had intact motor function (Figure 6A). First, we examined anxiety‐like behaviours by performing open field, light–dark box tests, and elevated plus maze. The anxious mice tended to avoid open, exposed and brightly illuminated areas. The results demonstrated that *Arid1a* cKO mice spent less time in the centre of the open field and showed less entrance in the first 5 min of a 30‐min open field test compared to WT (Figure 6B). In light–dark box tests, cKO mice tended to spend less time (Figure 6C, left) and enter fewer times (Figure 6C, middle) in the light area, but spent much more time to enter the light box (Figure 6C, right). Moreover, exploration analysis of cKO mice showed less significant entries of open arm in an elevated plus maze than that of WT mice (Figure 6D, left), however, the time in the open arms showed unchanged (Figure 6D, right).

Then we conducted learning and memory tests by using Y maze experiment and Morris Water Maze. The results showed that *Arid1a* loss did not cause any difference in both tests as compared with WT (Figure S2 A–D). We also conducted contextual and cued fear conditioning, and the results showed there was no obvious difference between cKO and WT mice (Figure S2 E‐G). Marble burying test and self‐grooming were used to test autism‐like behaviour, but there was no difference in both tests (Figure S2 H‐I). Collectively, these results suggested that deletion of microglial *Arid1a* did not cause autism‐like behaviour or learning and memory deficits, but resulted in anxiety‐like behaviour.

## 4 DISCUSSION

Microglia are thought to play an essential role in the differentiation and maturation of precursors into neurons.^45^ However, the molecular mechanisms underlying the contribution of the phenotypic plasticity of microglia to neurogenesis remain largely unexplored. Here, we leverage microglia‐specific *Arid1a* deletion, which altered the dynamic equilibrium of microglial polarization states and caused defective neurogenesis and anxiety‐like behaviours, to gain mechanistic insights into the role of chromatin remodelling in the immune capacities of microglia during the development of CNS. This study elaborates the role of chromatin remodelling in governing microglia polarization and the effects of differentially polarized microglia on key functions of NSPCs during neurogenesis.

Classical M1‐ and M2‐polarization states originate from T‐helper cell type 1 (Th‐1) or T‐helper cell type 2 (Th‐2) that stimulated peripheral macrophages polarization.^46^ Microglia, regarded as CNS residing microphage, have two activation subtypes named M1 and M2, in conformity with the macrophage nomenclature.^47^, ^48^ Microglial polarization is one of the most significant features in a variety of CNS disorders such as traumatic brain injury, stroke and many atypical neurogenesis‐related diseases.^35^, ^49^, ^50^ In our analysis, we found that microglial M1 genes including *Ki*, *Tlr*, *Il1a*, *Il1b, Il6, Il18* and *Tmem47* significantly increased and M2 genes including *Igf1*, *C1qa, Arhgap22, Tim47, Timp2, sema6d* and *fgd2*, significantly changed. Among those M1 genes, aberrant expression of *Il1a, Il1b, Il6, Il18* lead to cells or brain damage,^51^ and *Tmem47* plays an important role in M1 microglial migration and could increase the aggregation of M1 microglia at the inflammatory site. M2 genes, including *sema6d* and *Igf1* play important roles in cell development,^52^, ^53^ *C1qa* is necessary for microglial phagocytosis,^54^ *fgd2* acts as a regulator in leukocyte signalling,^55^ which indicates that Arid1a is involved in microglia/microphage polarization balance.

Emerging evidence shows that chromatin remodelling plays an important role in microphage/microglia polarization.^56^, ^57^, ^58^ For example, a histone 3 Lys27 (H3K27) demethylase named Jumonji domain‐containing 3 (JMJD3), could activate M2 macrophage development.^59^ On the other hand, H3K4me3 enrichment on the promoter of M1 polarization markers, such as IL‐6, IL‐18, MCP‐1 and TNF‐α induces long‐term inflammation.^60^ H3K27ac and H3K4me3 are considered as transcription activation markers,^61^, ^62^ and the enrichment of H3K27ac and H3K4me3 on promoters leads to chromatin openness. Previous studies show that ARID1A is involved in H3K27ac modulation and chromatin accessibility.^23^, ^62^ Our results further support an essential role of chromatin openness in microglia/microphage polarization. However, whether *Arid1a*‐mediated chromatin accessibility crosstalk with these histone modifications in microglia needs further investigation.

In the mouse CNS, *Arid1a* is highly expressed in astrocytes, neurons and microglia,^63^ and lack of *Arid1a* in mouse NSPCs or human embryonic stem cells (hESCs) resulted in anomalous neurogenesis, accompanied by an increase of chromatin accessibility enrichment.^24^, ^28^ Our use of ATAC‐seq allowed us to connect microglia chromatin accessibility to the abnormal polarization pattern of M1/M2 microglia in *Arid1a* cKO mice. This connection, to our knowledge, has not been previously made. Recent studies have shown that polarized microglia have differential effects on the differentiation potential of NSPCs, with M1 microglia inhibiting neurogenesis, while M2 microglia supporting neurogenesis.^35^ By showing that M1 microglial polarization was enhanced, but M2 polarization was weakened following microglia‐specific *Arid1a* deletion, we observed impairment of neuronal differentiation, but not proliferation, and this is mainly due to Il1a expression enhancement upon *Arid1a* loss. Although Il1a has been reported to stimulate proliferation of NSPCs, however, in our analysis, we found that the expression of Il6, previously demonstrated to inhibit proliferation of NSPCs, was increased after *Arid1a* loss in microglia. Therefore, the concurrent increase of Il1a and Il6 expression after *Arid1a* loss may lead to no effects on NSPCs proliferation.^64^

Increasing evidence suggests that immune cells play important roles in brain development and behaviours. Defects of immune cells, including CD4^+^ T‐cells, Th17/Treg, mast cell and microglia,^41^, ^42^, ^43^, ^44^ lead to anxiety or anxiety‐like behaviours. Recent studies have shown that stimulated M2 polarization intervention enhances neurogenesis and rescues brain damage including brain injury, anxiety and stroke.^65^, ^66^, ^67^, ^68^ In agreement with an important role of microglia polarization in neurogenesis, our study revealed unbalanced switch of M1/M2 microglial polarization following microglia‐specific *Arid1a* deletion, which resulted in deficient neurogenesis both in vivo and in vitro. Il4, regarded as microglial M2 polarization inducible factor, can switch microglial M1/M2 polarization and alleviate neurological damage.^69^ Unlike M1 microglia that requires constant stimulation, a single pulsed stimulus with Il4 is sufficient to maintain M2 microglia polarization.^35^ In our study, Il4‐stimulated M1/M2 polarization switch rescued neurogenesis deficiency induced by *Arid1a* absence in microglia. This is consistent with a recent study in which IL4 overexpression specifically in microglia enhances neurogenesis, in which Il4‐stimulated WT microglia can increase both Tuj1+ and GFAP+ cells significantly,^35^ and protects mice from stress‐induced depressive‐like behaviours.^70^ Moreover, it has been reported that adult‐born neurons are required to buffer stress responses and depressive behaviour,^71^ suggesting that enhancement of adult neurogenesis can relieve symptoms of mental illness. Our findings that deficient neurogenesis and anxiety‐like behaviours following microglial *Arid1a* deletion was rescued by Il4 stimulation in vivo opened a new avenue of mitigating depressive illness through manipulation of microglial polarization states.

Our data suggest that cellular secretion process, such as cytokine secretion from microglia plays a critical role in modulating neurogenesis. But cellular secretion is a very complicated process. In addition to cytokines, metabolite, microRNA and extracellular vesicle also play irreplaceable role in cell‐to‐cell mutual regulation.^72^, ^73^, ^74^ One limitation of our study is that we did not examine the effects of metabolites in microglia on neurogenesis. Recent studies have shown that chromatin modifications are intimately tied to metabolic processes.^75^ and energy metabolism. Subunits of the SWI/SNF complex are originally identified as transcriptional regulators of genes involved in growth in the presence of alternative fermentable carbon sources, such as sucrose.^76^, ^77^ Mammalian SWI/SNF complexes are a family of polybromo‐associated BAF complexes, and the subunit Baf60c regulates glycolytic metabolism.^78^ Moreover, increasing evidence suggests that metabolic reprogramming plays a key role in innate inflammatory response. Switch of metabolic programme from a growth‐encouraging capacity (M2) to a killing/inhibitory capacity (M1) permits macrophages to respond with appropriate functions under specific conditions.^79^, ^80^ For example, when stimulated with LPS, microglia switch from oxidative metabolism toward glycolytic metabolism.^81^ Based on our RNA‐seq analysis, we found that there was a functional weakness in metabolic biosynthesis (KEGG: mmu00601, mmu00515, and mmu00562, date not shown), and the cKO mice showed a significant body weight loss, which is consistent with previous reports showing that *Arid1a* regulates glutathione and lipid metabolism.^82^, ^83^ To extend our findings, future study on the connection between chromatin remodelling, microglia polarization and metabolism is needed.

In conclusion, our studies uncover an unforeseen link between chromatin remodelling, dynamic equilibrium of microglial polarization, and defective neurogenesis and anxiety‐like behaviours. Reprogramming microglia polarization states with IL4 can restore deficient neurogenesis caused by *Arid1a* loss in microglia. These results provide a mechanistic insight into the role of chromatin remodelling in microglial biology, and highlight a new direction of mitigating neuropsychiatric disorders through manipulation of the activity of chromatin remodelers.

## AUTHOR CONTRIBUTIONS

Maolei Gong and Chang‐Mei Liu, conception and design, collection and assembly of data, data analysis and interpretation, manuscript writing, final approval of manuscript; Xiao Liu, Ruoxi Shi, Yijun Liu, Jinpeng Ke, and Hong‐Zhen Du, collection and assembly of data.

## CONFLICT OF INTEREST

The authors declare no conflict of interest.

## Supporting information


**Figure S1** **(**A, B). Schematic of modified microglial *Arid1a*. The Cx3cr1‐cre (A) or Cx3cr1‐creERT2 (B) mice were crossed to *Arid1a* ^*fl/fl*^ mice, in which irreversible knockout of Arid1a expression is induced upon Cre recombinase expression in microglia cells. (C,D) Number of microglia in cortex and hippocampus in *Arid1a* ^*fl/fl*^;Cx3cr1‐creERT2 mice line. Iba1 was used for frozen section staining at P14 mice (C) and quantified in cortex (D) and hippocampus (E). (F) Correlation heatmap of RNA‐seq (*N* = 3, F, left) and ATAC‐seq (*N* = 2, right). (G). Plotted of transcription start site (TSS) and transcription end site (TES) of NCBI‐annotated genes in ATAC‐seq. (H) Genome‐browser view of ATAC‐seq at Il6, *N* = 2. (I). Brdu staining of neural stem/progenitor cells (NSPCs). 100,000 NSPCs from WT mice were co‐cultured with 20,000 cKO or WT microglia, BrdU was added into the culture systems 6 h before staining of BrdU and Iba1. (J) Proliferation analysis of NSPCs. (K) Apoptosis test of NSPCs in vitro. NSPCs and microglia were cultured as in I for 24 h, TUNEL and Iba1 were used for NSPCs apoptosis test. (L) Quantification of apoptosis NSPCs. Scale bar = 50 μm. Data are expressed as mean ± SEM and analysed by Student's *t*‐test from at least three independent experiments. **p < 0.05 and ****p < 0.0001.
**Figure S2.** Unimpaired behaviour in selective deletion of *Arid1a* in microglia. (A) The spontaneous alternation of WT and cKO mice in Y maze. (B) Freezing level (percentage) of WT and cKO mice for contextual fear conditioning. (C) Cued fear memory after the indicated fear conditioning. (D) Learning curves of WT and cKO mice in MWM tests with hidden platform. (E–G). In probe trials on Day 6, there was no significant difference in time of target crossings (E), number of target crossings (F) and latency to locate the platform (G) between the two groups of mice. (H). Quantification of buried beads. (I). Self‐grooming behavioural test. (J). Body weight of WT and cKO mice. Data are expressed as mean ± SEM and analysed by Student's *t‐*test, *n* ≥ 12, ****p < 0.0001.
**Table S1:** Primers for qRT‐PCR
**Tabel S2:** Genotyping primers

## Data Availability

The RNA‐seq and ATAC‐seq datasets generated and analyzed in this study have been deposited in the NCBI Sequence Read Archive (SRA). The raw data for microglia RNA‐seq on p15 extracted using FACS are accessible through the series accession number PRJNA812340 (https://www.ncbi.nlm.nih.gov/bioproject/PRJNA812340/). For primary microglia ATAC‐seq are accessible through the series accession number PRJNA 812630 (https://www.ncbi.nlm.nih.gov/bioproject/PRJNA812630/). For mouse Arid1a ChIP‐seq accessible through the series accession number PRJNA638150 (https://www.ncbi.nlm.nih.gov/bioproject/PRJNA638150/).
