# Supplementary material for: Abnormal microglial polarization induced by Arid1a deletion leads to neuronal differentiation deficits
Source: Cell Prolif. 2022 Jul 19;55(11):e13314. doi: 10.1111/cpr.13314 (PMC9628249; doi:10.1111/cpr.13314)
Supplement: Supplementary file 1 — Figure S1 (A, B). Schematic of modified microglial Arid1a. The Cx3cr1‐cre (A) or Cx3cr1‐creERT2 (B) mice were crossed to Arid1a fl/fl mice, in which irreversible knockout of Arid1a expression is induced upon Cre recombinase expression in microglia cells. (C,D) Number of microglia in cortex and hippocampus in Arid1a fl/fl ;Cx3cr1‐creERT2 mice line. Iba1 was used for frozen section staining at P14 mice (C) and quantified in cortex (D) and hippocampus (E). (F) Correlation heatmap of RNA‐seq (N = 3, F, left) and ATAC‐seq (N = 2, right). (G). Plotted of transcription start site (TSS) and transcription end site (TES) of NCBI‐annotated genes in ATAC‐seq. (H) Genome‐browser view of ATAC‐seq at Il6, N = 2. (I). Brdu staining of neural stem/progenitor cells (NSPCs). 100,000 NSPCs from WT mice were co‐cultured with 20,000 cKO or WT microglia, BrdU was added into the culture systems 6 h before staining of BrdU and Iba1. (J) Proliferation analysis of NSPCs. (K) Apoptosis test of NSPCs in vitro. NSPCs and microglia were cultured as in I for 24 h, TUNEL and Iba1 were used for NSPCs apoptosis test. (L) Quantification of apoptosis NSPCs. Scale bar = 50 μm. Data are expressed as mean ± SEM and analysed by Student's t‐test from at least three independent experiments. **p < 0.05 and ****p < 0.0001. Figure S2. Unimpaired behaviour in selective deletion of Arid1a in microglia. (A) The spontaneous alternation of WT and cKO mice in Y maze. (B) Freezing level (percentage) of WT and cKO mice for contextual fear conditioning. (C) Cued fear memory after the indicated fear conditioning. (D) Learning curves of WT and cKO mice in MWM tests with hidden platform. (E–G). In probe trials on Day 6, there was no significant difference in time of target crossings (E), number of target crossings (F) and latency to locate the platform (G) between the two groups of mice. (H). Quantification of buried beads. (I). Self‐grooming behavioural test. (J). Body weight of WT and cKO mice. Data are expressed as mean [file CPR-55-e13314-s001.docx]

**Abnormal Microglial Polarization Induced by Arid1a Deletion Leads to Neuronal Differentiation Deficits**

Maolei Gong^1,3,4^, Ruoxi Shi^1,2,3,4^, Yijun Liu^1,2,3,4^, Jinpeng Ke^1,2,3,4^, Xiao Liu^1, 3,4^, Hong-Zhen Du^1,3,4^, Chang-Mei Liu^1,2,3,4*^

^1^State Key Laboratory of Stem Cell and Reproductive Biology, Institute of Zoology, Chinese Academy of Sciences, Beijing, China

^2^Savaid Medical School, University of Chinese Academy of Sciences, Beijing 100049, China

^3^Institute for Stem Cell and Regeneration, Chinese Academy of Sciences, Beijing 100101, China

^4^Beijing Institute for Stem Cell and Regenerative Medicine, Beijing, China

* Correspondence author: Chang-Mei Liu ([liuchm@ioz.ac.cn](mailto:liuchm@ioz.ac.cn), 86-10-82619690)

**SUPPLEMENTAL FIGURE LEGEND**

**
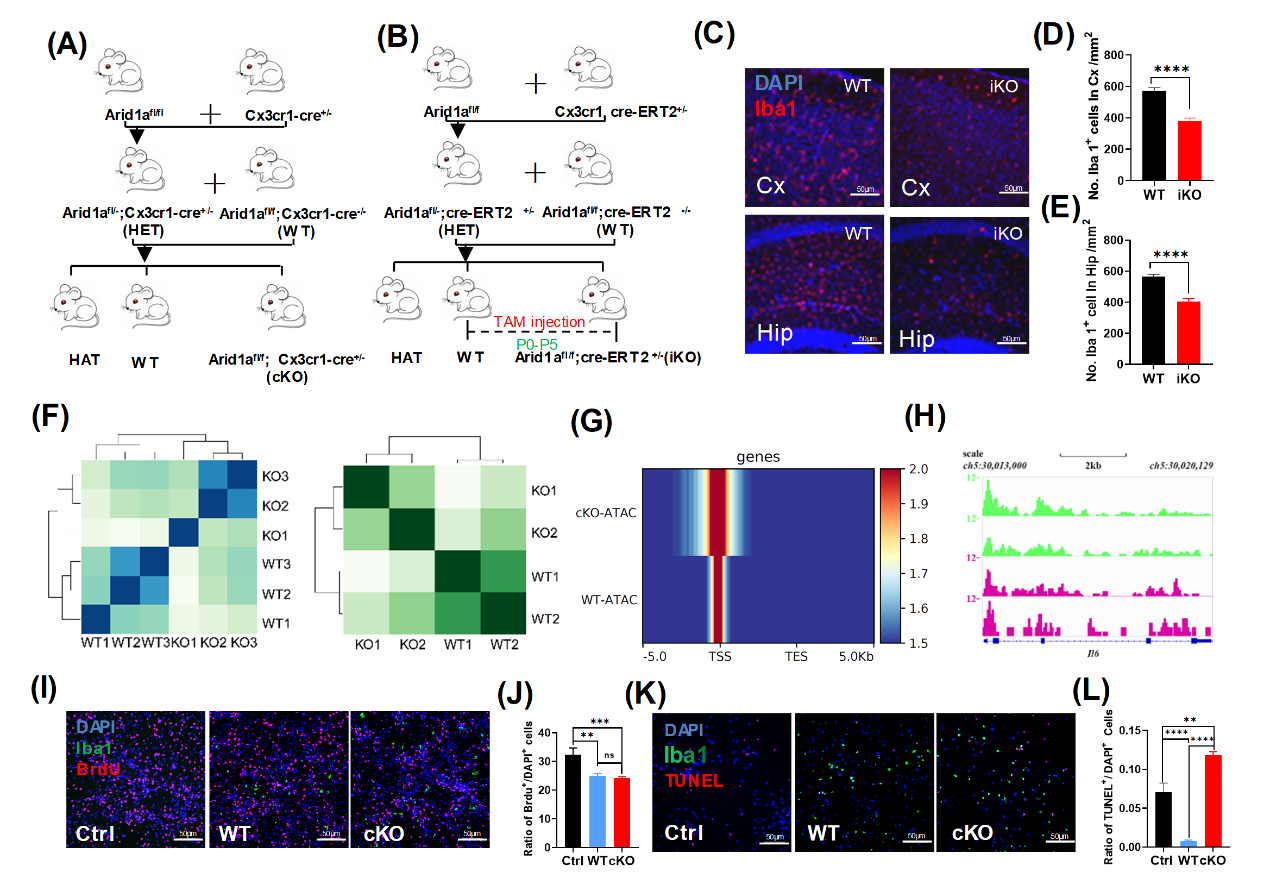
**

**Figure S1.** **(**A-B). Schematic of modified microglial *Arid1a*. The Cx3cr1-cre (A) or Cx3cr1-creERT2 (B) mice were crossed to *Arid1a^fl/fl^* mice, in which irreversible knockout of Arid1a expression is induced upon Cre recombinase expression in microglia cells. (C-D). Number of microglia in cortex and hippocampus in *Arid1a^fl/fl^*;Cx3cr1-creERT2 mice line. Iba1 was used for frozen section staining at P14 mice (C) and quantified in cortex (D) and hippocampus (E). (F). Correlation heatmap of RNA-seq (N=3, F, left) and ATAC-seq (N=2, right). (G). Plotted of transcription start site (TSS) and transcription end site (TES) of NCBI-annotated genes in ATAC-seq. (H). Genome-browser view of ATAC-seq at Il6, N = 2. (I). Brdu staining of NSPCs. 100,000 NSPCs from WT mice were co-cultured with 20,000 cKO or WT microglia, BrdU was added into the culture systems 6 h before staining of BrdU and Iba1. (J). Proliferation analysis of NSPCs. (K). Apoptosis test of NSPCs in vitro. NSPCs and microglia were cultured as in I for 24 hr, TUNEL and Iba1 were used for NSPCs apoptosis test. (L). Quantification of apoptosis NSPCs. Scale bar= 50μm. Data are expressed as mean ± SEM and analyzed by Student's T Test from at least three independent experiments. ***p<0.05* and *****p < 0.0001*.


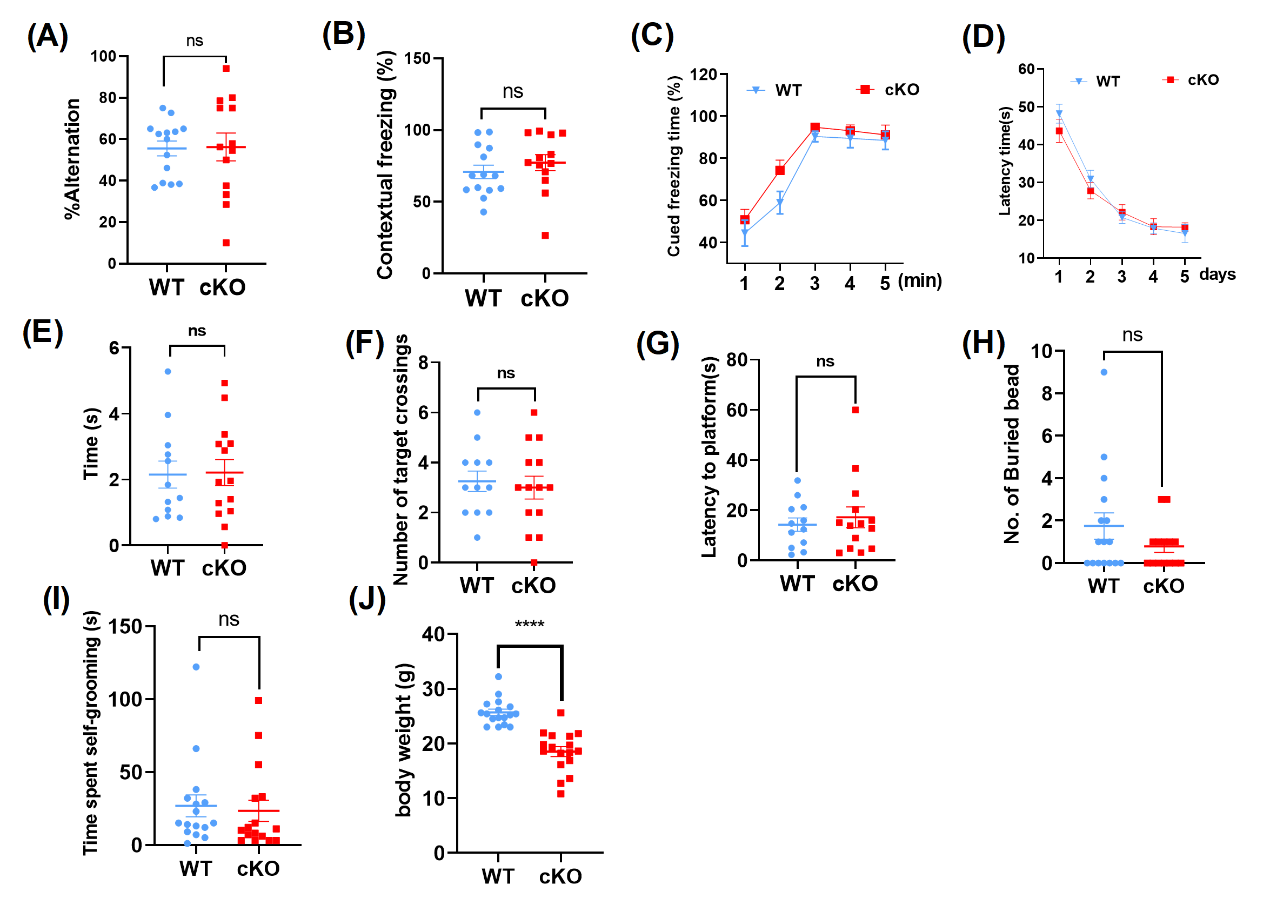


**Figure S 2. Unimpaired behavior in selective deletion of *Arid1a* in microglia.** (A). The spontaneous alternation of WT and cKO mice in Y maze. (B). Freezing level (percentage) of WT and cKO mice for contextual fear conditioning. (C). Cued fear memory after the indicated fear conditioning. (D). Learning curves of WT and cKO mice in MWM tests with hidden platform. (E-G). In probe trials on Day 6, there was no significant difference in time of target crossings (E), number of target crossings (F) and latency to locate the platform (G) between the two groups of mice. (H). Quantification of buried beads. (I). Self-grooming behavioral test. (J). Body weight of WT and cKO mice. Data are expressed as mean ± SEM and analyzed by Student's T, n≥12, *****p < 0.0001*.

**Supplementary Table legends**

**Table 1: Primers for qRT-PCR**

| **Genes** | **Forward Primer (5' -> 3')** | **Reverse Primer (5' -> 3')** |
| --- | --- | --- |
| *Arg1* | CTCCAAGCCAAAGTCCTTAGAG | GGAGCTGTCATTAGGGACATCA |
| *Arhgap2* | CCTTCGCTGCCAGATACTTCA | CCCTCGTAGCACAAACCATCG |
| *Arid1a* | CTTCCCCAACCACCAGTACAA | CTGTGCGAAGGACGAAGAC |
| *Atp8a2* | ACCAACAGGACGGTACACCA | TCTTTCCACATGATAGTGTGCC |
| *Bhlhe41* | ATGTGTAAACCCAAAAGGAGCTT | TCGGGCAGTAAATCTTTCAGC |
| *C1qa* | CCAGGAGAGTCCATACCAGAA | GTCCCACTTGGAGATCACTTG |
| *Cd34* | ATCCCCATCAGTTCCTACCAAT | TGGTGTGGTCTTACTGCTGTC |
| *Cx3cr1* | TCTGGACTCACTACCTCATCAG | TCCGGTTGTTCATGGAGTTGG |
| *Fgd2* | CCACGCCCAGTTCTTCCTTC | GCTGAGACTTGTCCATCCACG |
| *Igf1* | CACATCATGTCGTCTTCACACC | GGAAGCAACACTCATCCACAATG |
| *Il18* | GACTCTTGCGTCAACTTCAAGG | CAGGCTGTCTTTTGTCAACGA |
| *Il1a* | TCTCAGATTCACAACTGTTCGTG | AGAAAATGAGGTCGGTCTCACTA |
| *Il1b* | GAAATGCCACCTTTTGACAGTG | TGGATGCTCTCATCAGGACAG |
| *Il6* | TCTATACCACTTCACAAGTCGGA | GAATTGCCATTGCACAACTCTTT |
| *Kl* | GGGACACTTTCACCCATCACT | ACGTTGTTGTAACTATCGCTGG |
| *Sema6d* | CCCGTTTGATGCCCGACAA | GGAAGTGTGGTTCTTTGATCCA |
| *Serpine2* | ACATGGGATCGCGTCCATC | GCCACGGTCACAATGTCTTT |
| *Siglech* | GTCCTACCAATGTGATGTGCTC | CCCAGGGATATGAGGGCAG |
| *Timp2* | CTGGACGTTGGAGGAAAGAAG | CTGGGTGATGCTAAGCGTGTC |
| *Tmem119* | CCTACTCTGTGTCACTCCCG | CACGTACTGCCGGAAGAAATC |
| *Tmem47* | TGGCATTGCGAATCCACTCTC | CGTGATCCCACACAGATTGAAAT |
| *Trim47* | CGAGGTGATGGGGTTCATCG | AGCTGACTGAATCCGCCTCT |
| *Ttr* | CTGCTGTAGACGTGGCTGTAA | CTTCCAGTACGATTTGGTGTCC |

**Tabel 2: Genotyping Primers**

| Genes | Primer names | **Primer (5' -> 3')** |
| --- | --- | --- |
| *Arid1a* | Flox-F | TGG GCA GGA AAG AGT AAT GG |
|  | Flox-R | AAC ACC ACT TTC CCA TAG GC |
| *CX3CR1cre* | Common-F | CTG TTG CCT CAA CCC CTT TA |
|  | WT- R | TCT GGG TTC CTA GTG GAG CTA |
|  | Mut- R | CAC CTT CCT CTT CTT CTT GGG |
| *CX3CR1cre-ERT2* | *Common-F* | *AAG ACT CAC GTG GAC CTG CT* |
|  | *WT- R* | *CGG TTA TTC AAC TTG CAC CA* |
|  | *Mut- R* | *AGG ATG TTG ACT TCC GAG TTG* |
